# Experimental and Numerical Analysis of Initial Plasticity in P91 Steel Small Punch Creep Samples

**DOI:** 10.1007/s11340-017-0296-9

**Published:** 2017-05-19

**Authors:** F. Cortellino, J. P. Rouse, B. Cacciapuoti, W. Sun, T. H. Hyde

**Affiliations:** Department of Mechanical, Materials and Manufacturing Engineering, University of Nottingham, Nottingham, Nottinghamshire NG7 2RD UK

**Keywords:** Small punch test, Creep, Finite element, Initial plastic strain

## Abstract

To date, the complex behaviour of small punch creep test (SPCT) specimens has not been completely understood, making the test hard to numerically model and the data difficult to interpret. This paper presents a novel numerical model able to generate results that match the experimental findings. For the first time, pre-strained uniaxial creep test data of a P91 steel at 600 ^∘^C have been implemented in a conveniently modified Liu and Murakami creep damage model in order to simulate the effects of the initial localised plasticity on the subsequent creep response of a small punch creep test specimen. Finite element (FE) results, in terms of creep displacement rate and time to failure, obtained by the modified Liu and Murakami model are in good agreement with experimental small punch creep test data. The rupture times obtained by the FE calculations which make use of the non-modified creep damage model are one order of magnitude shorter than those obtained by using the modified constitutive model. Although further investigation is needed, this novel approach has confirmed that the effects of initial localised plasticity, taking place in the early stages of small punch creep test, cannot be neglected. The new results, obtained by using the modified constitutive model, show a significant improvement with respect to those obtained by a ’state of the art’ creep damage constitutive model (the Liu and Murakami constitutive model) both in terms of minimum load-line displacement rate and time to rupture. The new modelling method will potentially lead to improved capability for SPCT data interpretation.

## Introduction

In recent years, miniature specimen testing techniques have received increased attention from the power generation industry in view of several situations where the amount of material available for testing is limited [1, 2]. One of these cases is life assessment of in-service components, where small amounts of material can be extracted without impairing the structural integrity of the analysed component [3, 4]. In this way, quasi-non destructive evaluation of “at risk” components can be completed to inform future operation and maintenance strategies. The uncertainty related to life assessment techniques needs to be reduced if safe and economic operation is to be achieved and in-service component testing constitutes a key approach [7]. Miniature specimen creep testing techniques developed for power plant applications and, in particular for material creep properties characterisation, include: subsize conventional uniaxial creep specimens, with typical diameters of 1.5 to 3 mm; impression creep test specimens, with typical dimensions of 10x10x2.5 mm for a rectangular sample; small punch creep test specimens, with recommended disk radius of 8 mm and thickness of 0.3 to 0.5 mm; small ring creep test specimens, with typical radius of 6 mm; and two-bars creep specimens, with typical overall length of 26 mm [2, 5, 6].

The small punch creep test (SPCT) involves the application of a constant load through a hemispherical end indenter on a small disk. The small punch testing technique was first developed in 1980s for nuclear applications in order to evaluate the ductile-to-brittle transition temperature (DBTT) of a Ni-Cr steel (as a constant load-line displacement rate test technique). In 1990s, the creep deformation determined by this testing technique, carried out at constant load, was used to characterise the creep properties of various steels and of their welds.

An application of small specimen testing techniques is the creep qualification of critical locations in structural components, such as the heat affected zone (HAZ) of welds [7–9].

Typically, the test has been used for power plant applications (e.g. steam headers or steam pipes) testing materials such as P91, P92, CrMoV steels, 316LN stainless steel, and magnesium and aluminium alloys [10–13]. Typical temperatures are those of the creep regime of the material been tested, e.g. 575–750 ℃.

SPCT has several advantages over other miniature testing techniques. Specimen designs are simple (a disk) and therefore far easier to manufacture than, say, the miniature samples proposed by the Electrical Power Research Institute (EPRI) [1]. Unlike other creep samples, such as the small ring specimen [2], the small punch creep test specimen is taken to failure, therefore it is expected that creep rupture data can also be evaluated using SPCT [14–22].

During a unixial creep test, carried out at constant stress, crack propagation only occurs in the tertiary region of the creep curve. During small punch creep tests however, damage starts developing in the secondary stage of the deflection versus time curve, where deformation of the specimen is dominated by dislocation creep. The tertiary region of the deflection versus time curve is characterised by an acceleration of the crack propagation and the deformation of the disk is governed by inter-granular cavitation [23, 24]. A small punch specimen generally experiences the evolution of a necking region, which takes place at an offset from the axis of symmetry of the disk [25–27]. Commonly, a crack nucleates at the bottom surface of the specimen, in the necked area, and spreads through the thickness of the disk, leading to the specimen rupture [3, 28].

The complex, non-linear nature of the test introduces several difficulties in the procedure of data interpretation and correlation to the corresponding uniaxial test output. In particular, large plastic deformation due to the application of the loading punch takes place in localised regions of the specimen. It is expected that this deformation affects both the localised creep behaviour of the material in those regions and the global deformation response of the specimen as a whole.

Several authors have investigated the effects of plasticity (induced in a material by various loading conditions) on subsequent creep deformation [29]. Willis et al. investigated the effects of pre-straining on the creep response of 316H stainless steel in partially-solution treated (PST) and fully-solution treated (FST) conditions, and of FST 316L stainless steel, at 575 ^∘^C [30, 31]. A drastic reduction in the creep ductility and the minimum creep rate was observed for both PST and FST 316 stainless steels, while the failure time was found to decrease for the PST material and to increase for the FST material. A similar variation of the creep curve, after room temperature pre-straining, was also observed by Wilshire and Palmer for polycrystalline copper [32]. Wilshire and Willis also reported the effects of high temperature pre-straining on the subsequent creep behaviour of 316L stainless steel at 575 ^∘^C by reducing the test stress after an initial stage. Immediately after the load reduction, the creep rate became extremely low and, after that, it increased to a value similar to the minimum creep strain rate observed during a constant stress creep test [31].

The behaviour of 316 stainless steel was also investigated by Hyde who reported the results of plasticity/creep tests performed at 550 ^∘^C by applying a creep load to the specimens and overloading for 3 sec at regular time intervals of 168 h [33, 34].

In view of the remarkable effects that initial plasticity (pre-straining) can have on the creep behaviour of materials, the role of initial, large plastic deformation on the response of the small punch creep test specimen is investigated in this paper through the use of finite element (FE) calculations and experimental investigations carried out on both full sized uniaxial and small punch specimens. For the first time in the published literature, the data obtained from pre-strained uniaxial creep tests has been used in numerical analyses in order to simulate the effects of the initial plasticity on small punch creep test, resulting in a good agreement between the numerical results and the experimental data. Liu and Murakami’s creep damage model [35] has been conveniently modified in order to take into account, during numerical simulations of small punch creep tests, the effects of pre-straining. The modified creep damage model has shown to be able to provide more accurate results in terms of displacement versus time curve with respect to the non-modified Liu and Murakami model. The results of the non-modified constitutive model exhibit times to failure one order of magnitude shorter than those obtained by experimental testing and numerical calculations carried out with the modified constitutive model.

## Experimental Programme

### Pre-strained Uniaxial Creep Tests

#### Experimental Procedure

**Fig. 1:**
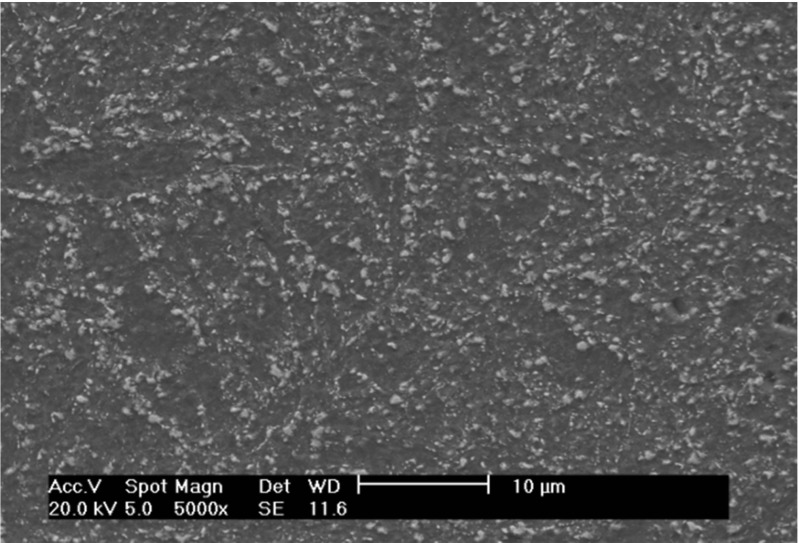
SEM image of the virgin P91 steel used for the tests, from Ref. [37]

The testing programme consisted of pre-strained uniaxial creep tests and SPCTs carried out on a P91 steel at 600 ^∘^. Table 1 reports the chemical composition, in wt%, of the P91 steel used in the present work, while Fig. 1 shows an SEM image of the virgin material microstructure. Cylindrical uniaxial specimens were machined on a CNC lathe from a power plant steam pipe section. The data obtained from the pre-strained uniaxial creep tests was used to characterise the plastic behaviour of the material through the determination of two parameters, *ϕ* and *ψ*, as described in Section 2.1.3.

**Table 1:** Chemical composition (wt%) of the P91 steel used for the investigation [36]

Cr	Mo	C	Si	S	P	Al	V	Nb	N	W	Fe
8.60	1.02	0.12	0.34	< 0.002	0.017	0.007	0.24	0.070	0.060	0.03	Bal

**Fig. 2:**
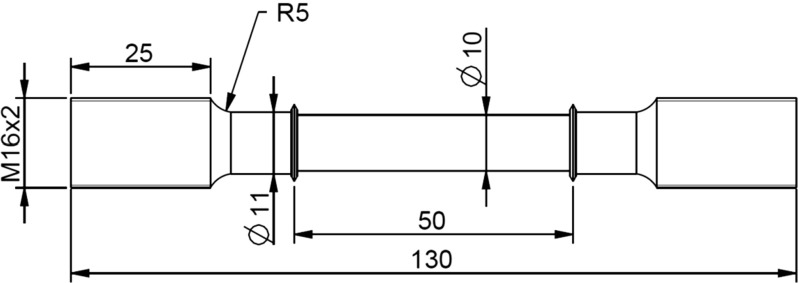
Uniaxial creep test specimen (dimensions in mm) used to perform pre-strained creep tests

Before manufacturing the specimen (see Fig. 2 for the specimen geometry) material blanks were tempered at 760 ^∘^C for 3 hours and then cooled to room temperature at a rate of 0.8 ^∘^C/min , in order to obtain a fully martensitic steel microstructure.

A Mayes EN250 machine was used for all of the pre-strained uniaxial creep tests. The deformation of the specimen was monitored by use of an extensometer with a gauge length of 50 *mm*, while the temperature was kept constant to within ± 1 ^∘^C by the furnace controller of the machine. For each test, the specimen was plastically pre-strained under displacement control conditions. Subsequently, the configuration of the machine was changed to load control and constant load creep tests were carried out. The load levels used during the creep tests were corrected in order to account for the reduction of the cross sectional area due to plastic pre-straining. When plastic deformation takes place the volume of the specimen is constant, therefore equation (1) holds, where Ω_*pre*_ and *L*_*pre*_ are the cross section area and the gauge length of the specimen at the end of pre-straining, while Ω_*yield*_ and *L*_*yield*_ are the cross section area and the specimen gauge length at yielding.

1 $$ {\Omega}_{pre}L_{pre}={\Omega}_{yield}L_{yield}  $$

The load, *P*, applied to the specimen is then obtained by equation (2), where *σ* is the stress applied, *d*_0_ is the initial diameter of the specimen’s effective section, *𝜖*_*el*,*yield*_ is the elastic engineering strain at yielding, *𝜖*_*p*_ is the plastic pre-strain and *ν* is the Poisson’s ratio. Deviation of equation (2) is presented in the Appendix.

2 $$ P=\sigma{\Omega}_{pre}=\sigma\pi\frac{{d_{0}}^{2}}{4}\frac{1+\epsilon_{el,yield}}{1+\epsilon_{p}+\epsilon_{el,yield}}\left( 1-\nu\epsilon_{el,yield}\right)^{2}  $$

The stress levels used for the creep tests are 150, 160 and 170 *M* *P* *a*. For the 150 *M* *P* *a* tests, engineering pre-strain levels of 5, 10 and 20*%* were used. For the other two stress levels, the pre-strain levels of 0.5, 5, 10 and 20*%* were used. Uniaxial creep data at 600 ^∘^C was also available for the P91 steel and was used to provide 0*%* pre-strain creep results [36].

#### Pre-strained creep test results

**Fig. 3:**
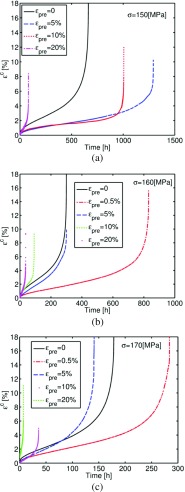
Variation of the creep strain, *𝜖* ^*c*^, versus time (**a**) 150 *M* *P* *a*, (**b**) 160 *M* *P* *a* and (**c**) 170 *M* *P* *a* at 600 ^∘^C, where *𝜖*_*pre*_ is the total engineering pre-strain

Figure 3 shows the variation of the creep strain, *𝜖* ^*c*^, versus time obtained from the pre-strained creep tests performed at 150, 160 and 170 *M* *P* *a* at 600 ^∘^C, where *𝜖*_*pre*_ is the total engineering pre-strain. All of the plots exhibit the typical three regions of creep curves, i.e. primary, secondary and tertiary regions, and the experimental output was found to be significantly influenced by the plastic pre-straining carried out before creep testing. Plastic deformation mainly takes place in the pre-straining stage of the test, as the creep step is carried at stress levels well below the material’s yield stress (in it’s virgin state). The effects of plastic strain accumulation in the latter stages of the test (i.e. just before rupture) are not considered hereafter.

The results of the tests performed at 150 *M* *P* *a* (Fig. 3(a)), show that the material exhibited a creep resistance effect, i.e. the minimum creep strain rate decreased and the time to failure increased, for pre-strain levels of 5 and 10*%*, while, with a pre-strain level of 20*%*, a creep enhancement effect was found (i.e. the minimum creep rate increased and the time to rupture decreased). When the stress level was 160*M* *P* *a*, see Fig. 3(b), creep resistance effects were found for pre-strains of 0.5 and 5*%*, and creep enhancement was observed for 10 and 20*%* pre-straining. For the tests at 170*M* *P* *a*, the material showed creep resistance effects for 0.5*%* pre-strain only, while, for all of the other cases, creep enhancement occurred, as reported in Fig. 3 (c).

#### Characterisation of the effects of pre-straining on creep response

In view of the results shown in Fig. 3, the effects of high temperature plastic pre-strain on creep response of P91 steel may be quantified by two parameters, namely, *ϕ* and *ψ*. These parameters account for the variation in the minimum creep strain rate and the time to failure, respectively, due to the application of a total engineering pre-strain level, *𝜖*_*t*_. Equations 3 and 4 defines the *ϕ* and *ψ* parameters, respectively, where ${\dot {\epsilon }}^{c}_{MIN,0}$ and *t*_*f*,0_ are the minimum creep strain rate and the failure time of material under a 0*%* pre-strain condition (as received), while ${\dot {\epsilon }}^{c}_{MIN,p}$ and *t*_*f*,*p*_ refer to the material after pre-straining.

3 $$ \phi=\frac{{\dot{\epsilon}}^{c}_{MIN,p}}{{\dot{\epsilon}}^{c}_{MIN,0}}  $$

4 $$ \psi=\frac{t_{f,0}}{t_{f,p}}  $$

In the experimental study conducted by Tai and Endo [38], the *ϕ* parameter was used to model the variation of the minimum strain rate after pre-creep deformation in a 2.25Cr1Mo steel. In the present work the accumulation of time dependent inelastic strain during pre-straining is considered to be negligible.

For each stress level, the variation of the *ϕ* parameter with *𝜖*_*p*_ was fitted by an equation of the form of equation (5) (see Table 2 for coefficient values) where *𝜖*_*p*_ is the plastic engineering pre-strain, expressed as a percentage. The values of each fitting coefficient, obtained for each stress level, was averaged over the three stress levels, in order to obtain an average fitting surface.

5 $$ \phi=exp[{a_{1}\epsilon_{p}}]+{b_{1}{\epsilon_{p}^{2}}}+{c_{1}{\epsilon_{p}^{3}}}+{d_{1}{\epsilon_{p}^{4}}}  $$

**Table 2:** Averaged fitting constants values used in equation (5)

*a*_1_	*b*_1_	*c*_1_	*d*_1_
−5.9534	6.690x 10^−2^	−8.800x 10^−3^	3.236x 10^−4^

**Fig. 4:**
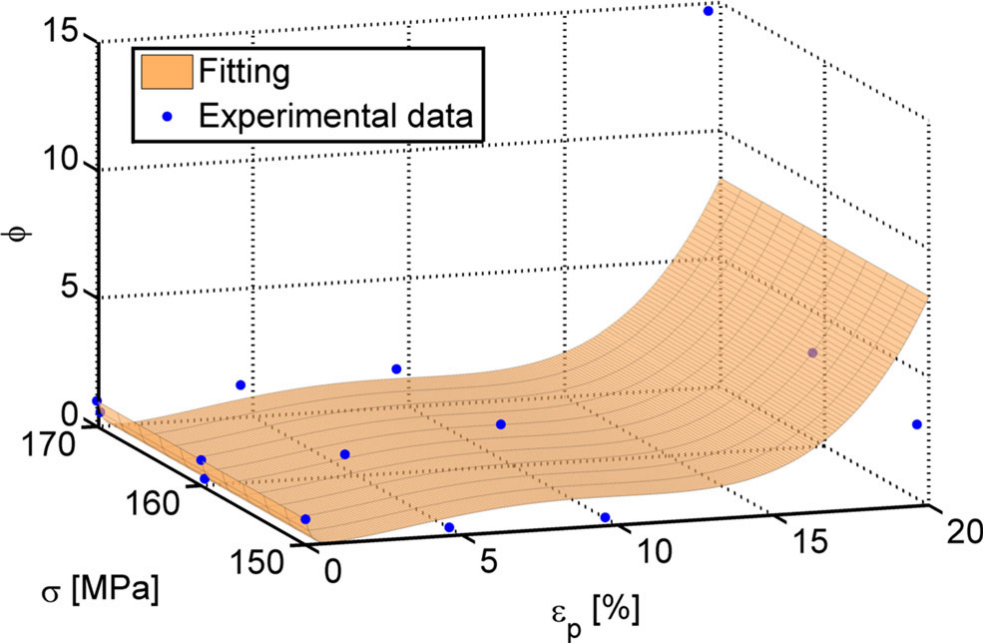
Variation of the *ϕ* parameter with the engineering plastic pre-strain and the stress level with an average fitted surface to the experimental data

Figure 4 shows the *ϕ* parameter versus the engineering plastic pre-strain and the stress (for creep) obtained from the experiments, together with the average fitting surface (see equation (5)).

Similarly, the rupture data for the pre-strained creep tests was used to determine the variation of the *ψ* parameter. Equation 6 is the fitting equation used to model the *ψ* variation versus *𝜖*_*p*_, for each stress level, and Table 3 lists the coefficients of the average fitting.

6 $$ \psi=exp[{a_{2}\epsilon_{p}}]+{b_{2}{\epsilon_{p}^{2}}}+{c_{2}{\epsilon_{p}^{3}}}  $$

**Table 3:** Averaged fitting constants for equation (6)

*a*_2_	*b*_2_	*c*_2_
-3.241	2.610x 10^−2^	4.763x 10^−4^

**Fig. 5:**
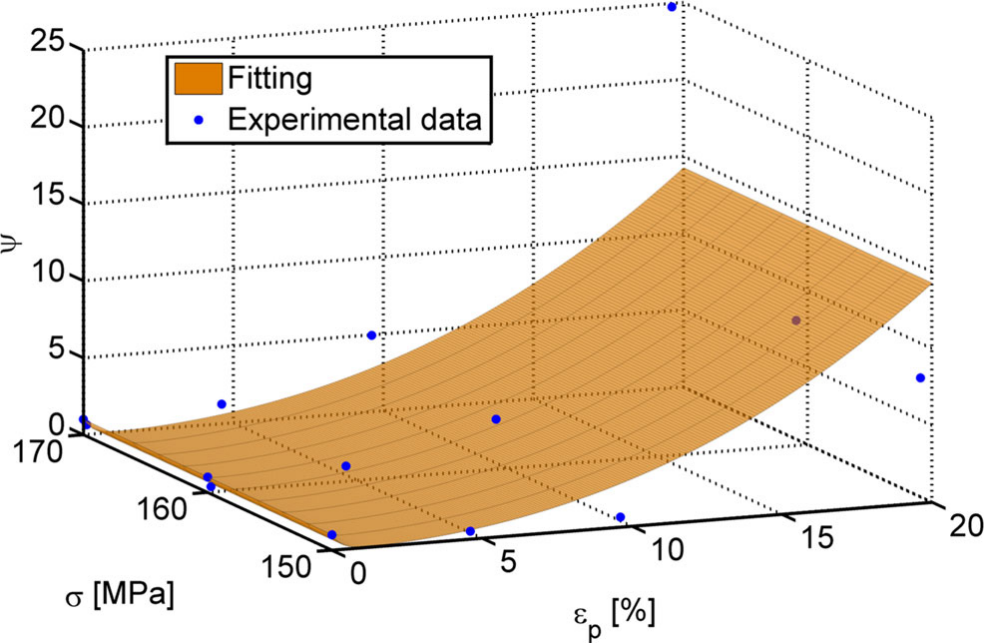
Variation of the *ψ* parameter with the engineering plastic pre-strain and the stress level with the average fitted surface to the experimental data

Figure 5 shows the variation of the *ψ* parameter with the engineering plastic pre-strain and stress obtained from the experiments, and the average surface (equation (6)).

When *ϕ* > 1, the material shows an increase of the minimum creep strain rate when compared with the 0*%* pre-strained condition (hence it exhibits a creep enhancement effect). When *ϕ* < 1, a creep resistance effect is evident. Similarly, when *ψ* < 1 the failure life of the specimen increases (creep resistance effect) while, when *ψ* > 1, the failure time decreases (creep enhancement effect). Both equation (5 and 6) lead to *ϕ* = *ψ* = 1 when the plastic pre-strain equals 0, i.e. for the 0*%* pre-strained condition.

### Small Punch Creep Tests

#### Experimental Set-up

**Fig. 6:**
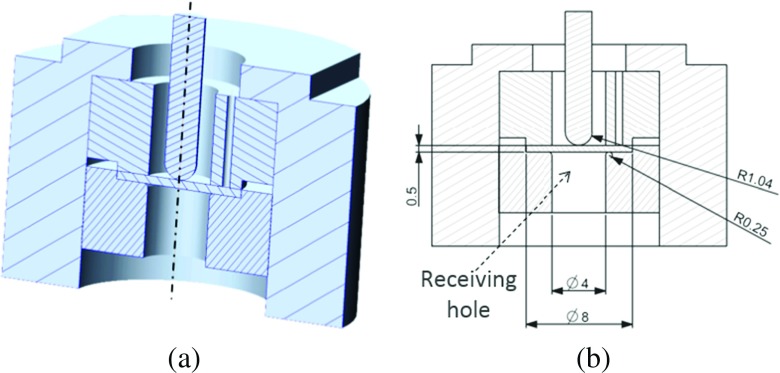
Specimen location set-up with relevant dimensions (mm)

Small punch creep tests were carried out at 600 ^∘^C on the same batch of material as that used for the pre-strained tests (see Table 1). A dead-weight machine was used for the SPCTs (see Fig. 6(a) for a cross section of the experimental set-up). The disc specimen was held in place by a clamping ring while it was loaded by the hemispherical end punch. The outer tube and the clamping ring have through-passing holes for the top thermocouple to be inserted. In Fig. 6(b), the dimensions of the testing set-up, which conform to those recommended by the CEN draft code of practice [5], are also shown.

The specimen was clamped by applying a clamping torque to a M25x1.5 thread, connecting the outer tube and the insert tube. The value of the clamping force was controlled by monitoring the applied torque and which is set to 10*N* *m* for all of the tests, in order to ensure consistent experimental conditions.

The set-up represented in Fig. 6 was located in a furnace with a single-zone temperature controller. During the tests, the temperature was controlled to 600 ± 1^∘^C. Two thermocouples were inserted in the testing set-up (located at the top surface of the specimen and immediately below the specimen) in order to record any temperature fluctuations.

The central deformation of the specimen was monitored by a linear variable differential transducer (LVDT), which measures the displacement of the punch with time.

To date, the magnitude of friction forces acting on SPCT specimen has not been identified in a definitive manner. However, numerical and experimental investigations [39–41] showed that, for the same test load and temperature, friction has the effect of increasing the time to rupture and decreasing the rate of creep deformation of the specimen. Friction forces generate a bending moment in the specimen which opposes the rotation of the annular region of the specimen in the vicinity of the edge of contact between the specimen and the punch. In turn, the viscous (creep) deformation of the specimen takes place at a lower rate and the failure time is therefore increased [40]. Although SPCT is a static test, the temperature and the test load level in the present investigation are likely to be high enough for the asperity junctions of the punch and the specimen to generate a stick/slip scenario. The generation of such contact regime is intrinsic of this testing typology, therefore, in the present work, it could not be eliminated.

#### Small punch creep test results

**Fig. 7:**
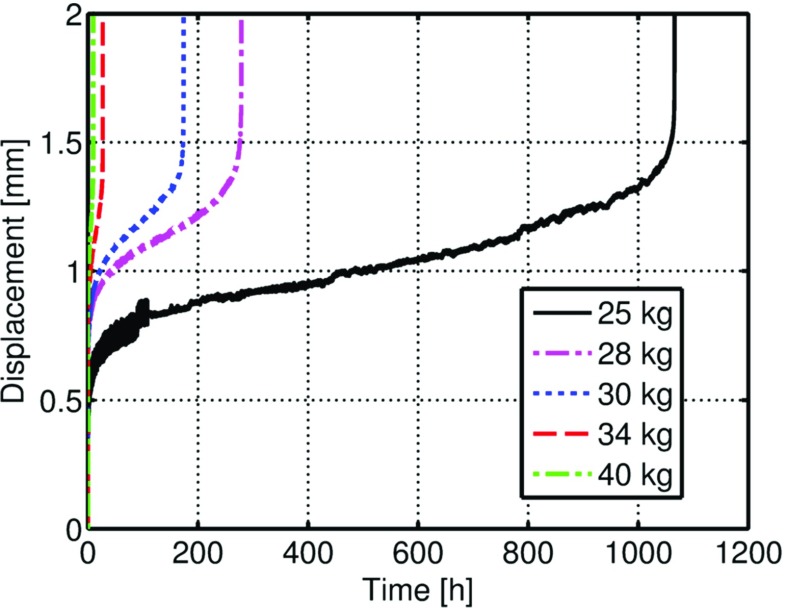
Variation of the central specimen deformation versus time for a P91 steel at 600 ^∘^ with different load levels

SPCTs were performed with punch loads (*P*) of 25, 28, 30, 34 and 40*k* *g*. Figure 7 shows the experimental output of the tests, i.e. the variation of the central displacement of the specimen versus time. The experimental curves shown in Fig. 7 exhibit the 3 typical SPCT regions described in the literature [1, 20, 42–44].

**Fig. 8:**
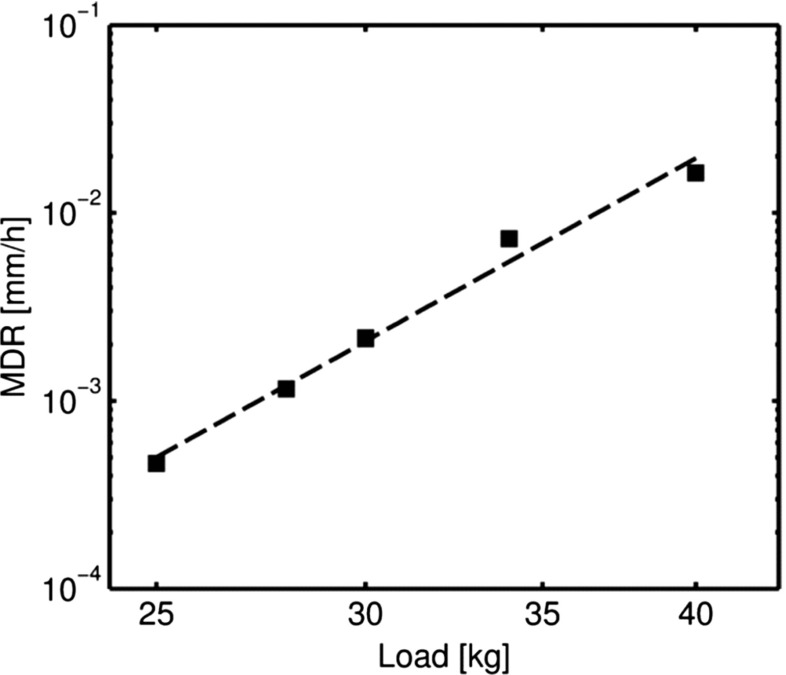
Variation of the *MDR* versus the load level, plotted on logarithmic scales

The early stage of the test is characterised by a decreasing displacement rate, followed by a stationary region, where the displacement rate reaches its minimum value. The later stage of the tests shows rapidly increasing displacement rate until failure occurs. The fluctuations in displacement found in the early stages of the 25*k* *g* test curve were caused by electronically induced noise, caused by interference between two data acquisition system units; this was eliminated in the early stages of the other tests. Figure 8 shows the variation of the minimum displacement rate (*MDR*) versus the load level, *P*.

A power law correlation between *MDR* and *P* was found and is represented by equation (7), where *MDR* is expressed in *m* *m*/*h* *r* and *P* in *kg*.

7 $$ MDR=6.457\text{x}10^{-15}P^{7.790}  $$

**Fig. 9:**
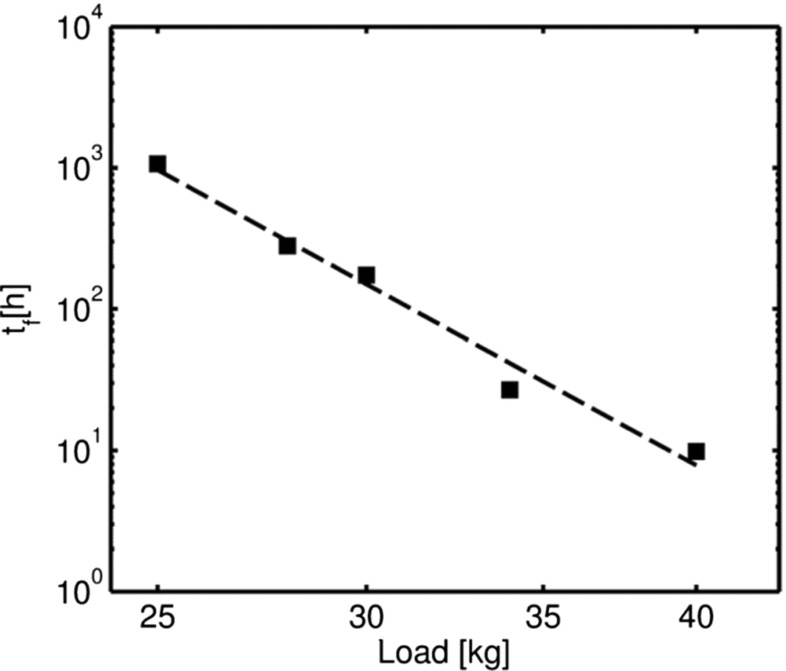
Variation of the time to failure (*t*_*f*_) versus load level, plotted on logarithmic scales

Figure 9 shows the variation of the failure time, *t*_*f*_, versus load level using logarithmic scales. A power law correlation of *t*_*f*_ with load level, given by equation (8), was obtained.

8 $$ t_{f}=1.995\text{x}10^{17}P^{-10.24}  $$

According to the CEN Code of Practice CWA 15627, the load level, *P*, must be calculated such that the small punch creep test specimen has the same time to failure as an uniaxial creep test specimen subjected to an equivalent uniaxial stress, ${\sigma _{UNI}^{EQ}}$ [5]. The dimensions of the testing set-up, i.e. *R*_*s*_ = 1.04*m* *m*, *a*_*p*_ = 2*m* *m* and *t*_0_ = 0.5*m* *m*, were used in equation (9) and the multiplication factor of 0.8 was included because the disc specimens were clamped into the experimental set-up [5], conforming with the CEN code of best practice for SPCT. The *k*_*sp*_ parameter is a correction factor describing the effects of the ductility of the tested material at different temperatures. *k*_*sp*_ has then been calculated for each load level and it has been found to increase with the load, in agreement with the results of Gülçimen and Hähner [8]. An average *k*_*sp*_ value (1.353) was found for the load range used in the investigation.

9 $$ \frac{P}{\sigma_{UNI}^{EQ}}=3.332k_{sp}a_{p}^{-0.2}R_{s}^{1.2}0.8  $$

**Fig. 10:**
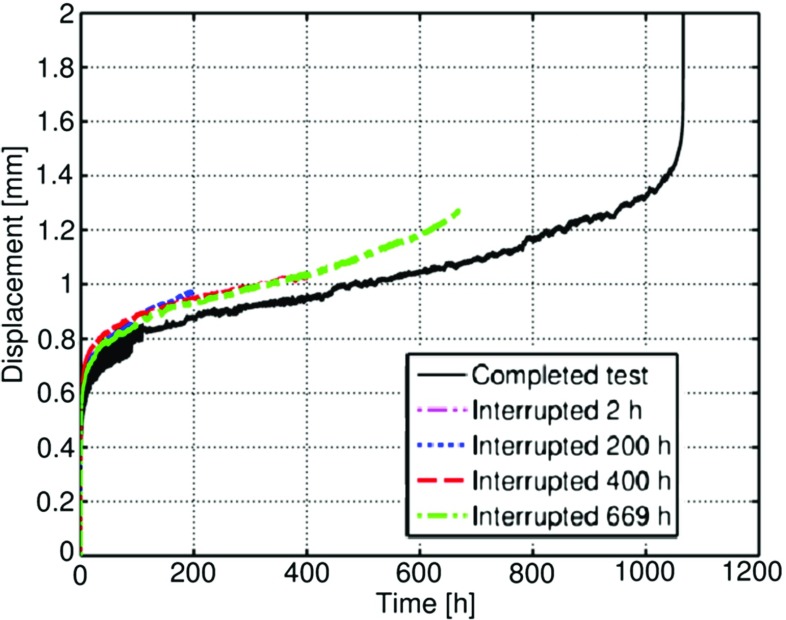
Variation of the central deformation of the specimen versus time for the interrupted tests. The punch load is 25*k* *g* for all of the tests

Interrupted tests were also performed using a load of 25*k* *g* in order to investigate the evolution of failure in the specimen during the test. Figure 10 is a plot of the output of the 25*k* *g* completed and interrupted tests (at 2, 200, 400 and 669 *hrs*). As with previous investigations, a significant amount of data scatter in the experimental results was found to exist [4]. In the present investigation, the scatter appeared to be related to the characteristics of the testing machine, and to slight misalignments in the loading punch with respect to the axial direction of the test rig. Detailed numerical investigations of this misalignment are discussed elsewhere [24, 45].

**Fig. 11:**
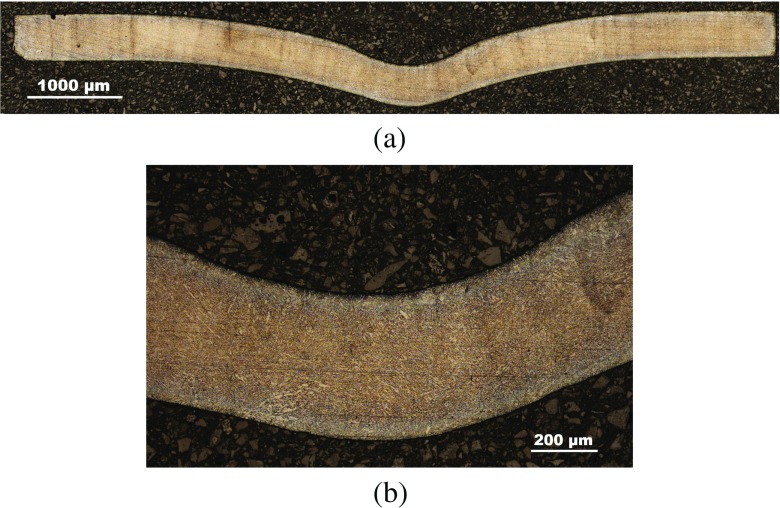
Images of a P91 SPCT specimen tested until 200*h* *r* *s*, showing (**a**) the general deformed shape and (**b**) detail of the punch contact region

**Fig. 12:**
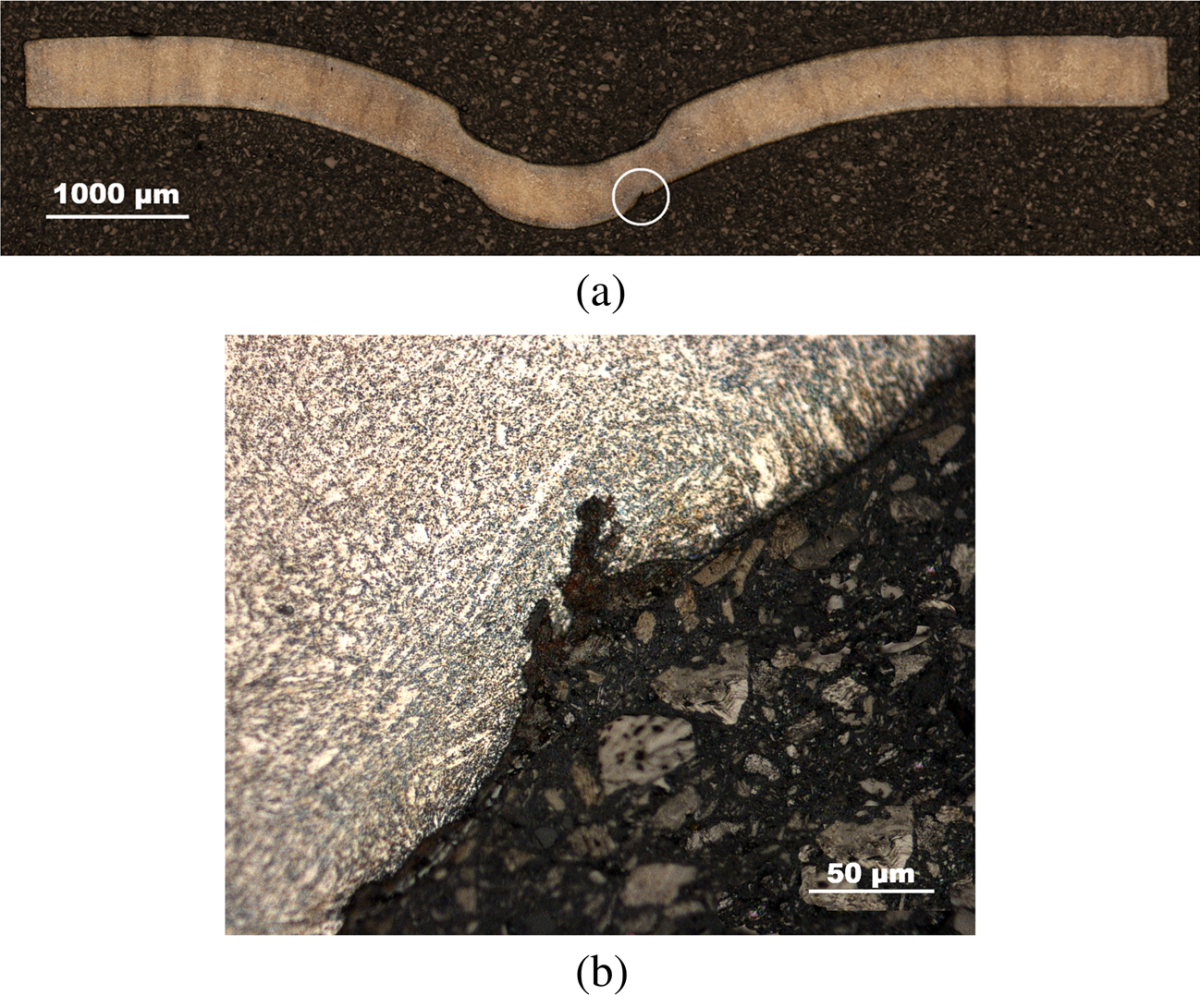
Images of a P91 SPCT specimen tested until 400*h* *r* *s*, showing (**a**) the general deformed shape and (**b**) detail of the crack initiated in the necking area

**Fig. 13:**
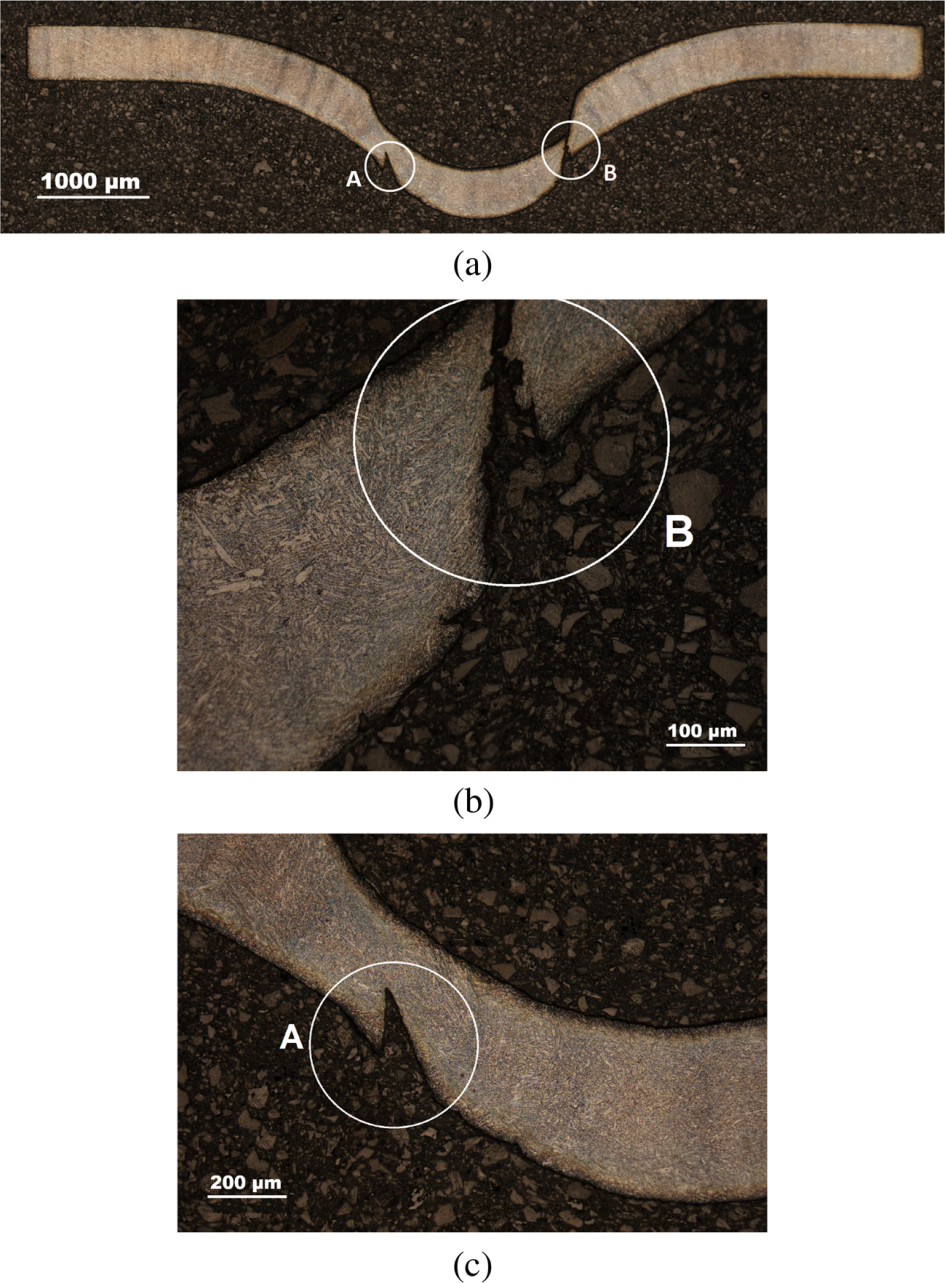
Images of a P91 SPCT specimen tested until 669*h* *r* *s* (failure), showing (**a**) the general deformed shape, (**b**) detail of the fracture surface (location B) and (**c**) detail of a secondary crack initiated in the punch contact region (location A)

Interrupted test specimens were imaged using Nikon Eclipse LV100ND optical microscope (see Figs. 11, 12 and 13). A crack was found to nucleate at the bottom surface of the specimen at some distance from the axis of symmetry in the 400*h* *r* *s* sample (see Figs. 12(b) and 13(c)) which propagates along a direction at approximately 45^∘^ from the loading axis (Fig. 13(b)).

Such a crack has a significant role in the evolution of the apparent creep response of the specimen, as it constitutes the main mechanism which leads to the loss of load carrying capability of the structure and to rupture (through fracture) of the specimen.

## FE Modelling

### FE Model

#### Material constitutive model

**Fig. 14:**
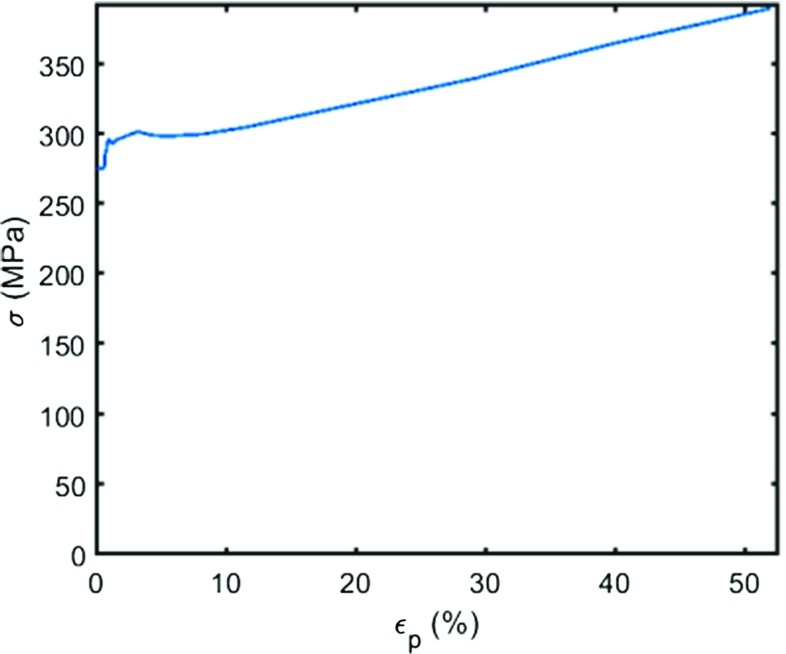
True stress/strain curve, for a P91 steel at 600 ^∘^C

An elastic/plastic/creep constitutive model, capable of accounting for the effects of plastic deformations on subsequent creep material behaviour, was used for the FE analyses. The true stress/strain curve, reported in Fig. 14, was approximated from the engineering tensile curve obtained using a stress/strain test of a P91 steel at 600 ^∘^C, and it was implemented, in a tabular form, in the FE solver ABAQUS (see Table 4), where *σ* is the equivalent von Mises stress and *𝜖*_*p*_ is the von Mises equivalent plastic strain.

**Table 4:** Tabular form of the true stress/strain curve, for a P91 steel at 600 ^∘^C, implemented in ABAQUS

*σ*(*M* *P* *a*)	*𝜖*_*p*_(*%*)
275.0	0.00
275.9	0.63
281.2	0.65
283.9	0.67
288.4	0.78
297.0	0.95
294.5	1.13
292.9	1.29
296.1	1.63
301.9	3.22
299.3	4.25
298.5	5.44
300.0	8.24
305.0	11.58
340.0	29.29
365.0	40.00
390.0	52.03

A creep damage model that takes into account the multiaxiality of the stress state, and thus it is suitable for numerical simulations of the small punch creep test, is the Liu and Murakami creep constitutive damage model [35]. Liu and Murakami based their model on those by Hutchinson and Riedel for materials undergoing creep-constrained grain boundaries cavitation [46, 47]. The former stated that a damaged material contains a dilute concentration of microcracks and, as a consequence, the creep strain rate has a linear dependence on the microcrack damage parameter [46]. Riedel extended Hutchinson’s model to non-dilute microcracks assumption, demonstrating the existing of an exponential relationship between the microcrack damage parameter and the creep strain rate for the uniaxial stress state [47]. In the Hutchinson model the creep strain rate is expressed as the sum of the dilatational creep components and the deviatoric creep components. According to Liu and Murakami, the former components are negligible when the material constant *n* is higher then 3 [35]. In the conventional damage mechanics, the damage parameter, *ω*, is intended as the ratio between the damaged area, due to material deterioration, and the initial undamaged area, and therefore it ranges from 0, for an undamaged material, to 1, for a failed/fractured material element. By assuming this definition for *ω* and by considering a cylindrical material cell, whose diameter and height have the same size, containing a penny shaped microcrack, or, equivalently, a cavitated grain boundary facet, Liu and Murakami proved that the damage parameter, *ω*, is a function of the length and the density of the microcracks and does not depend on the material constant *n* [35]. Instead the microcrack parameter, *ρ*, is also a function of *n*, as shown in equation (10) [35].

10 $$ \rho=\frac{2(n+1)}{\pi\sqrt{1+\frac{3}{n}}}\omega^{3/2}  $$

In this paper, in order to model the creep behaviour of the material by also taking into account the pre-straining effects in the SPCT, the authors propose a modified Liu-Murakami constitutive model, which was implemented through the CREEP user subroutine in ABAQUS [49]. The modified constitutive equations allow the effects of plastic pre-strain on the minimum creep rate and on the failure life to be included. The Liu-Murakami constitutive model was modified using the *ϕ* and *ψ* parameters defined in equation (3 and 4), respectively. Equations 11 and 12 shows the evolution of the creep strain rate, $\dot {\epsilon _{ij}^{c}}$, (in multi-axial form) and of the damage rate, $\dot {\omega }$, in the creep constitutive model adopted for the FE calculations, where *σ*_*EQ*_ is the von Mises equivalent stress, *σ*_1_ is the maximum principal stress, *S*_*ij*_ is the generic component of the deviatoric stress tensor and *B* is a material constant. *σ*_*RUP*_ is the rupture stress, defined by equation (13). The effects of temperature on the creep behaviour of the material are taken into account by the material constants. It should be noted that, in the Liu and Murakami constitutive model, the rupture stress is a composition of the maximum principal stress and of the von Mises equivalent stress, summarising the multi-axiality of the stress state in a single variable.

11 $$ \dot{\epsilon}^{c}_{ij}=\frac{3}{2}\phi{B}\sigma^{n}_{EQ}\left( \frac{S_{ij}}{\sigma_{EQ}}\right)exp\left[\frac{2(n+1)}{\pi\sqrt{1+\frac{3}{n}}}\left( \frac{\sigma_{1}}{\sigma_{EQ}}\right)^{2}\omega^{3/2}\right]  $$

12 $$ \dot{\omega}=\psi{A}\left[\frac{1-exp\left( -q_{2}\right)}{q_{2}}\right]\sigma^{\chi}_{RUP}exp\left( q_{2}\omega\right)  $$

13 $$ \sigma_{RUP}=\alpha\sigma_{1}+\left( 1-\alpha\right)\sigma_{EQ}  $$

In order to include the variations of *ϕ* and *ψ* with the plastic strain, equation (5 and 6) were also implemented in the constitutive model. The maximum damage value was limited to *ω*_*MAX*_ = 0.9901 in order to avoid numerical problems which arise when *ω* approaches unity.

In order to account for the loss of load carrying capability due to material creep deterioration, the creep constitutive model is fully coupled with the elastic material properties . The decrease in the stiffness of damaged elements with the increase of *ω* is governed by equation (14) [35], where *E*_0_ is the Young’s modulus of undamaged material, and *E* is the instantaneous modulus corresponding to a damaged element [48].

14 $$ E=E_{0}\left( 1-\omega\right)  $$

Table 5 lists the elastic properties and material constants for the Liu-Murakami creep constitutive model obtained for the P91 steel at 600 ^∘^C (as received 0*%* pre-strain condition).

**Table 5:** Material constants for a P91 steel at 600 ^∘^C (stress in *M* *P* *a* and time in *hrs*) adapted from ref. [36]

*E*_0_(*M* *P* *a*)	*ν*	*B*	*n*	*A*	*χ*	*q*_2_	*α*
1.5x 10^5^	0.3	1.51x 10^−30^	11.795	2.12x 10^−27^	10.953	5.3	0.3

**Fig. 15:**
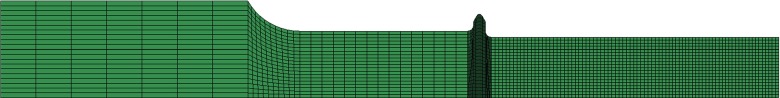
FE mesh used for the additional calculation of a uniaxial tensile test for a P91 steel at 600 ^∘^C

The correlations between pre-strain and the *ϕ* and *ψ* parameters were obtained using experimental results where strain levels are taken to be the engineering total strain for the specimen. In reality however, local strains (particularly in the necked regions) can be far greater than the average engineering strain of the uniaxial specimens (discussed in “Material constitutive model”). This is especially true for the high pre-strain level tests. In order to account for this mismatch and to implement a correlation between the true strains and the pre-strain parameters (i.e. *ϕ* and *ψ*) in the SPCT model, an additional FE calculation of a uniaxial tensile test of a P91 steel at 600 ^∘^C was carried out using the tensile curve described in Table 4. The mesh used is shown in Fig. 15 and it consists of 2974 axisymmetric quadrilateral elements. In view of the symmetry of the uniaxial creep test specimen (see Fig. 2), a quarter section only was modelled.

**Fig. 16:**
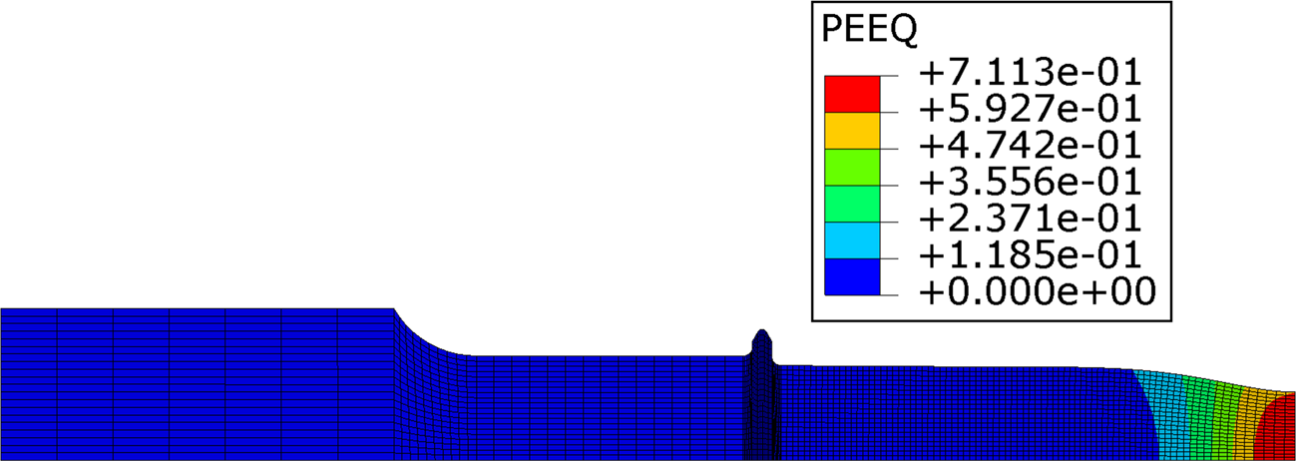
Contour plot of the equivalent plastic strain expressed in absolute value for an engineering strain (gauge length bulk average) of 0.122

**Fig. 17:**
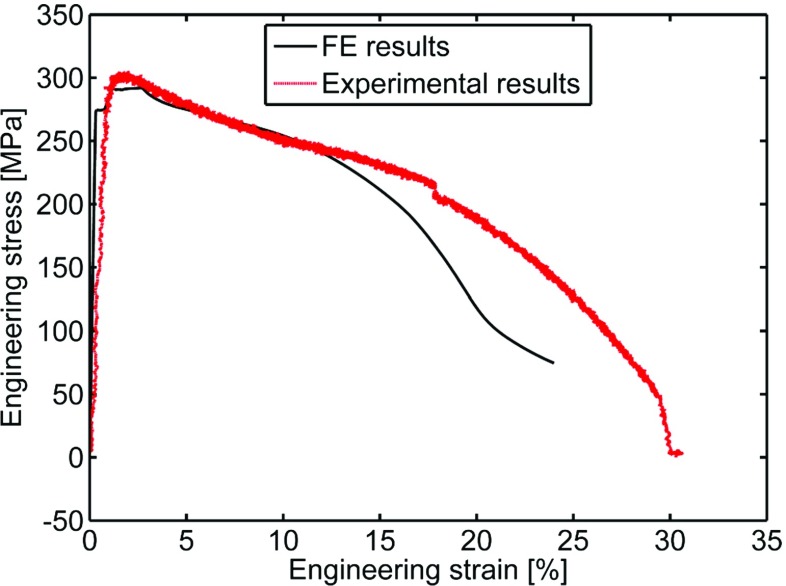
Experimental and numerical engineering stress/strain curves. Strain was measured using an extensometer with a gauge length of 50 *mm*. It is worth noting that the apparent “weakening” of the material is due to necking of the specimen rather than the degradation of the material

By using the geometry non-linear (GNL) approach (which updates the stiffness matrix of the structure at each time increment during the analysis) the uniaxial specimen FE model can take into account the large deformations and necking occurring for large engineering strains. Figure 16 is a contour plot of the plastic equivalent strain, expressed in absolute value, for an engineering strain level of 0.122*%*, while Fig. 17 shows the engineering stress/strain curves obtained from the uniaxial FE model and from a tensile test carried out on a P91 steel at 600 ^∘^C.

**Fig. 18:**
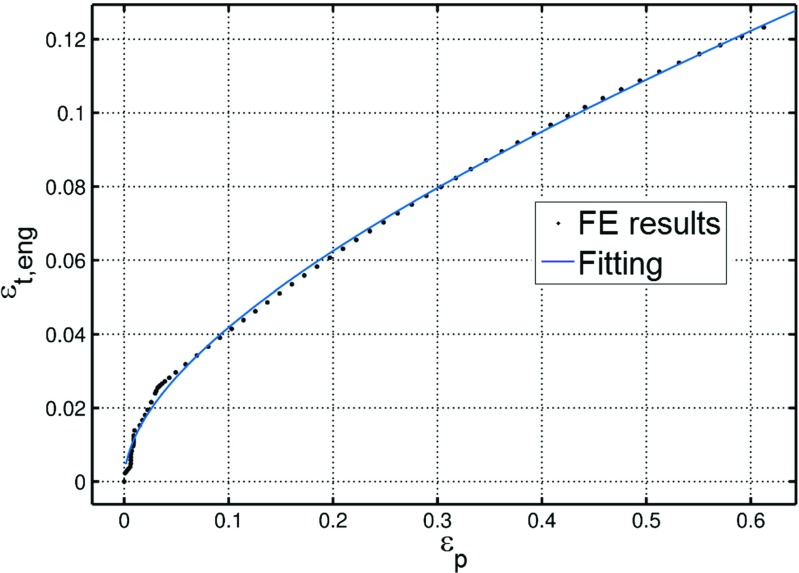
Variation of the engineering strain versus the true strain, in absolute value, obtained from the additional FE analysis of a tensile test for a P91 steel at 600 ^∘^C

From the results of the additional uniaxial FE analysis, the variation of the total engineering strain (*𝜖*_*t*,*eng*_) was related to that of the true equivalent plastic strain (*𝜖*_*p*,*true*_) in the necked section of the uniaxial specimen. Equation 15 was fitted to the FE results, where both *𝜖*_*t*,*eng*_, and *𝜖*_*p*,*true*_ are expressed in absolute value, with *a* = 0.052, *b* = 0.118 and *m* = 0.508 (Fig. 18).

15 $$ \epsilon_{eng}=a\epsilon_{true}+b\epsilon_{true}^{m}  $$

#### Geometry, loads and boundary conditions

The geometry of the FE model of the SPCT is identical to that used for testing as shown in Fig. 6, with a specimen thickness, *t*_*h*_, and diameter, *D*_*SPT*_, of 0.5*m* *m* and 8*m* *m*, respectively, punch radius, *R*_*s*_, of 1.04*m* *m*, receiving hole radius (see Fig. 6(b)), *a*_*p*_, of 2*m* *m* and lower clamp radius of 0.25*m* *m*.

**Fig. 19:**
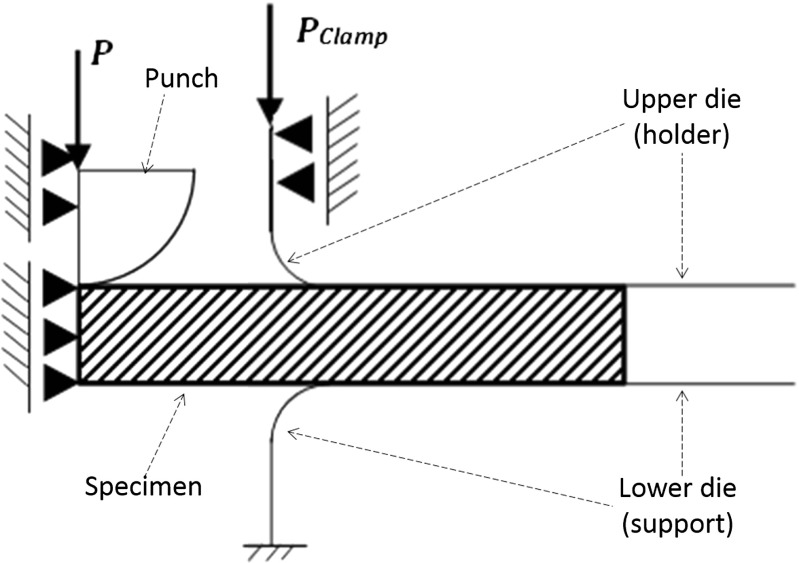
Loads and boundary conditions applied to the FE model of SPCT

Three load levels (25, 28 and 30*k* *g*) were applied to the punch in the analyses and suitable boundary conditions were imposed to the degrees of freedom of the rigid bodies which model the components of the test rig. The radial and axial translations and the rotation around the axis of symmetry of the support were constrained. The horizontal translation and the rotation of the punch and of the upper clamp were also constrained. A load of 500*k* *g* was applied to the rigid holder (representative of the clamping load resulting from a 10*N* *m* tightening torque [45]) in order to secure the specimen between the two dies. Figure 19 schematically shows the loads and the boundary conditions applied to the model.

#### Element choice and meshing

**Fig. 20:**
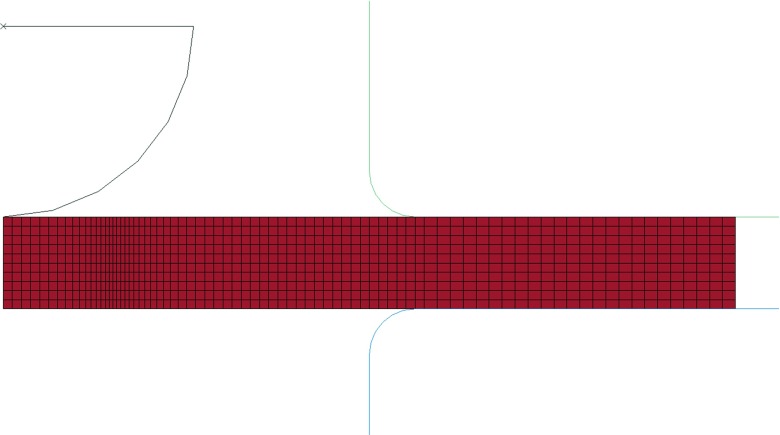
FE mesh used for the numerical simulations

Since the behaviour of the specimen is characterised by large incompressible deformations due to creep and by severe local loading conditions at the contact edge (between the punch and the specimen), the FE mesh adopted to model the SPCT specimen consists of 883 nodes and 790 bilinear axisymmetric 4-node elements. Figure 20 shows the mesh used for the SPCT FE analyses. 10 elements were used through the thickness of the specimen and the mesh was refined in the region where the edge of contact between the punch and the specimen is located for most of the analysis. The minimum element size, in the refined region, is 0.013 mm.

Quadratic elements (second order formulation) were avoided because, for the present work, they lead to a larger computational cost without producing any increase in the accuracy of the FE solutions as significant plastic deformations occur in the model. The reduced integration formulation was used, in order to avoid “locking” problems and convergence difficulties encountered when ”fully integrated” linear elements were adopted. These problems are due to a non-physical increase of the stiffness of the elements (“parasitic stiffness components” [49]) when bending deformation occurs, such as at the beginning of the creep step in the SPCT analyses, causing non-convergence of the solution. Hybrid formulation elements were adopted in order to avoid numerical singularities, which can occur when standard constant strain elements (such as the 4-node axis symmetric bilinear elements) are used with incompressible deformation fields. The hybrid formulation processes the pressure stress as an independently interpolated solution variable, coupled with the displacement solution [49]. Thus, unstable solutions for the displacement field after an infinitesimal increase of the pressure stress are avoided. In view of these requirements, the CAX4RH element type, available in ABAQUS, was used. The elements are refined near the edge of the unsupported region of the specimen, where significant bending deformation take place, and in the region close to the contact edge between the punch and the specimen, which was identified as the most critical location in the specimen [16, 28, 43].

#### Modelling procedure of the contact interactions

Surface-to-surface contact interactions have been used for all of the contacting pairs. The contact elements were automatically generated by the solver and consist of stiff springs which, once activated, apply the contact forces to the contact nodes on master and slave surfaces [50]. The activation of contact elements occurs when the interference between the contact nodes is less than the specified tolerance (0 in the present analyses). The stiffness of the contact elements varies non-linearly with the contact penetration (i.e. the non-linear penalty contact formulation was used). This was found to be a critical feature of the FE analyses when material flow due to plasticity and/or creep is included as it directly influences the convergence rate and the accuracy of the solution. Also the slip tolerance under stick conditions (identifying the stiffness of tangential contact elements) significantly affects the convergence rate of both the elastic/plastic and creep steps of the FE analysis. Although the friction coefficient between the specimen and the clamping components, as well as between the punch and the disk, cannot be straightforwardly quantified, for steels at temperatures of 600 ^∘^C or higher, a realistic value of the coefficient of friction between the punch and the specimen was found to be 0.3 by Dymáček et al. [41]. It was also demonstrated, by finite element analysis, that friction effects become important when the friction coefficient between the specimen and the punch varies from 0 to 0.5, making the failure life to increase up to 8 times [39, 40]. For this work the Coulomb classical friction formulation was used and the friction coefficients were taken to be 0.3 and 0.8 for the punch/specimen and the punch/clamps interactions, respectively [45]. The Coulomb friction formulation used for the numerical analyses does not account for stick/slip between contact surfaces. During the test the small disc specimen undergoes severe changes in its shape (it gradually deforms into a conical shape). In order to account for large deformation, the geometrical non-linearity formulation (GNL in ABAQUS) has been used for all of the analyses.

### Numerical Results and Discussion

**Fig. 21:**
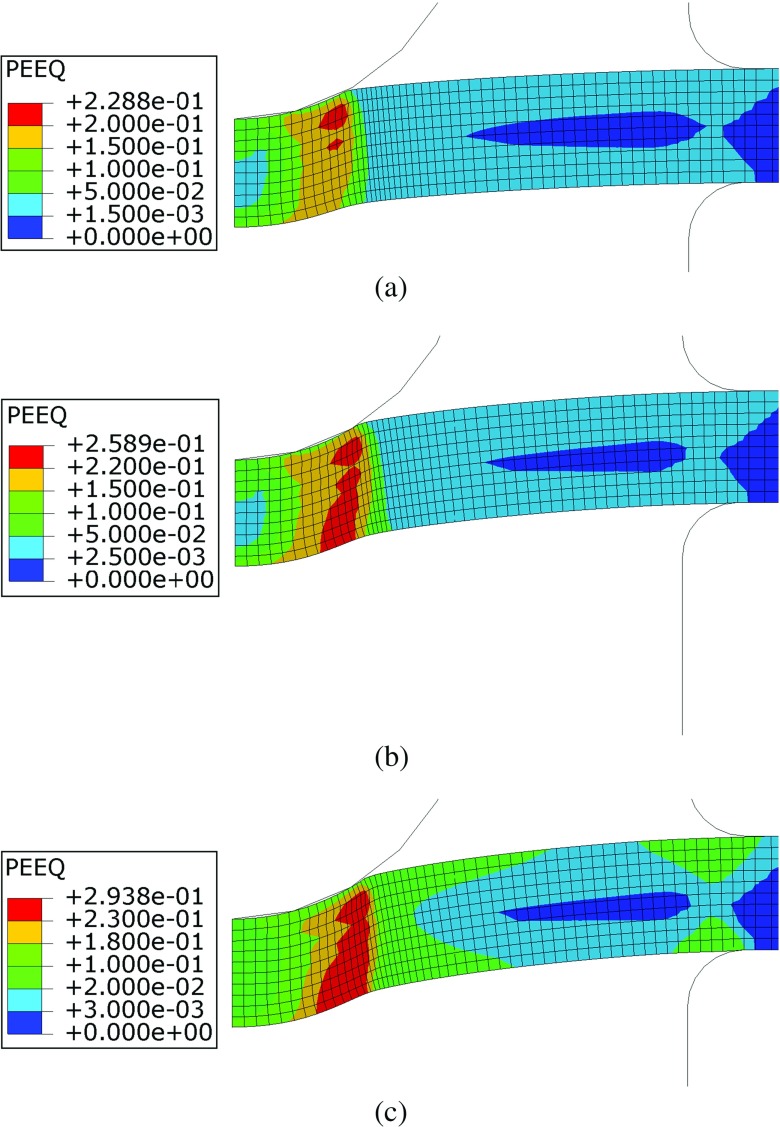
Contour plots of the equivalent plastic strains for punch loads of (**a**) 25*k* *g* (**b**) 28*k* *g* and (**c**) 30*k* *g* obtained at the beginning of the creep step of the analyses, i.e. *t* = 0*hrs*

The results of elastic/plastic/creep damage FE analyses of SPCTs obtained using the modified Liu-Murakami constitutive model (see equation (11 and 12)) were compared with those obtained without including the pre-straining effects (i.e. with *ϕ* = *ψ* = 1). Figure 21 shows the contour plots of the von Mises equivalent plastic strain at the beginning of the creep step. The von Mises peak plastic strains ranged from approximately 23*%* for a punch load of 25*k* *g* to approximately 29*%* when a punch load of 30*k* *g* was applied. The initial plastic strains are therefore judged to be significant in the specimen for all of the load levels used in the calculations. When the load increases from 25 to 30*k* *g*, the location of the peak equivalent plastic strain moves from the region close to the punch/specimen contact interface to the bottom surface of the specimen, at approximately 0.5*m* *m* from the axis of symmetry (see Fig. 21). When the load level is at the lower bound, at the beginning of the creep step, the deformation mechanisms of the specimen are bending and stretching, with high tensile stress and strain occurring at the bottom surface of the specimen in the region close to the punch/specimen contact edge. Furthermore, the punch locally loads the top surface of the specimen and generates, at a small depth under the contact surface, a localised stress field which induces a plastic strain level higher than that generated by global bending/stretching. The position of the peak stress and strain generated by contact is typical of that of an indentation problem, although the present configuration exhibits some differences from the ’classical’ Hertzian problem. When the load increases, stretching becomes more dominant over bending and the stress (and plastic deformation) it generates increases more than that generated by the contact interaction between the punch and the specimen, therefore, the location of the peak plastic strain changes with the load applied to the structure.

**Fig. 22:**
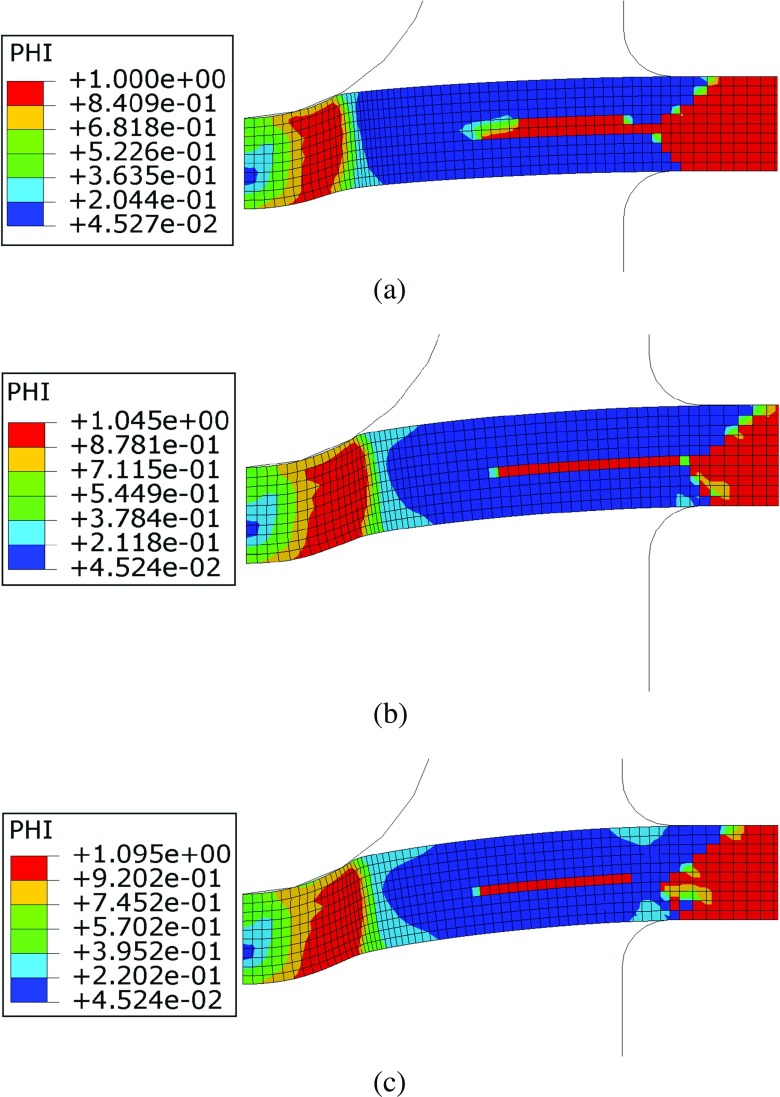
Contour plots of the *ϕ* parameter for punch loads of (**a**) 25*k* *g* (**b**) 28*k* *g* and (**c**) 30*k* *g*

**Fig. 23:**
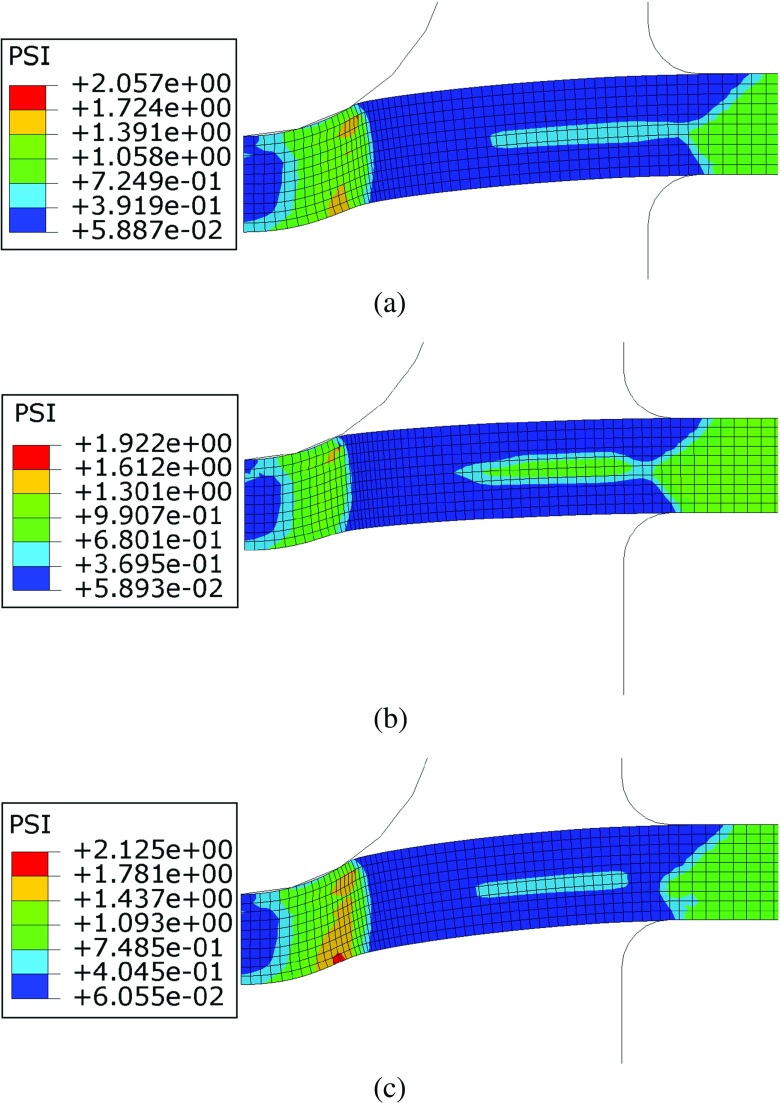
Contour plots of the *ψ* parameter for punch loads of (**a**) 25*k* *g* (**b**) 28*k* *g* and (**c**) 30*k* *g*

The effects of initial plastic deformations on the creep behaviour of the material are highlighted in Figs. 22 and 23, showing the contour plots of the *ϕ* and *ψ* parameters (calculated using the plastic strain levels shown in Fig. 21) at the beginning of the creep step, respectively.

In the region close to the punch/specimen contact edge (which is characterised by the peak plastic deformation) the *ϕ* parameter is 1 when the punch load is 25*k* *g*, while it is larger than unity (peak values are 1.095) for higher load levels. Therefore, a creep enhancement of the material occurs in that region for loads of 28 and 30*k* *g*. The region on the middle plane of the specimen, close to the clamps, exhibits *ϕ* = 1 for all of load levels used in the calculations because it corresponds to the neutral plane of bending deformation, where the material is not plastically deformed at the beginning of the test for any of the load levels implemented (see also Fig. 21 for comparison). The material surrounding this area of the specimen exhibits relatively small plastic strains, showing creep resistance effects (*ϕ* < 1) for all of the load levels.

**Fig. 24:**
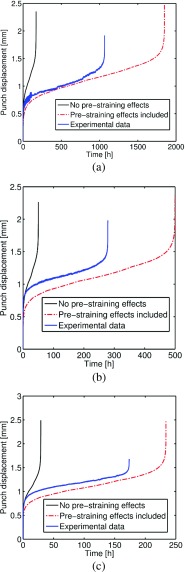
Variation of the punch displacement versus time with punch loads of (**a**) 25*k* *g* (**b**) 28*k* *g* and (**c**) 30*k* *g*

The variation of *ψ* shows that, due to plastic strain accumulation, the creep damage rate is enhanced in the area close to the punch/specimen contact edge (*ψ* > 1). A resistance effect is observed in the unsupported region (between the punch and the clamps where *ψ* < 1). In Fig. 23 the region of material on the middle plane of the specimen, close to the clamps, is characterised by *ψ* = 1 for all of the load levels because, in this region, *𝜖*_*p*_ = 0 (see Fig. 21). In the clamped region of the specimen the material does not experience any significant plastic deformation, therefore *ϕ* = *ψ* = 1. Figure 24 shows the punch displacement variation versus time obtained, for the three load levels, using both the modified (pre-strian effects) and un-modified constitutive model. These results are also compared to the corresponding experimental results (see Fig. 7).

From the results reported in Fig. 24 (where the effects of initial plastic deformation on the creep response of the specimen are taken into account) a global creep resistance effect can be observed with a significant decrease of the minimum displacement rate and an increase of the time to failure, compared to the results of the analyses obtained without the inclusion of pre-straining effects. The global creep resistance of the specimen is related to the fields of the *ϕ* and *ψ* parameters, which show values less than 1 in most of the specimen, except from the region close to the contact edge between the punch and the specimen. The results of the FE calculations performed by taking into account the effects of pre-straining exhibit a greater adherence to the experimental results compared to those obtained without modification in the creep damage constitutive model, in terms of both minimum displacement rate and time to failure. It should be noted that the results of the non-modified constitutive model exhibit times to failure one order of magnitude shorter than those obtained by experimental testing and numerical calculations carried out with the modified constitutive model. Albeit a more accurate quantification of the *ϕ* and *ψ* parameters could lead to a further improvement in the numerical results, the modification already provides a significant improvement with respect to the non-modified constitutive model calculations.

**Fig. 25:**
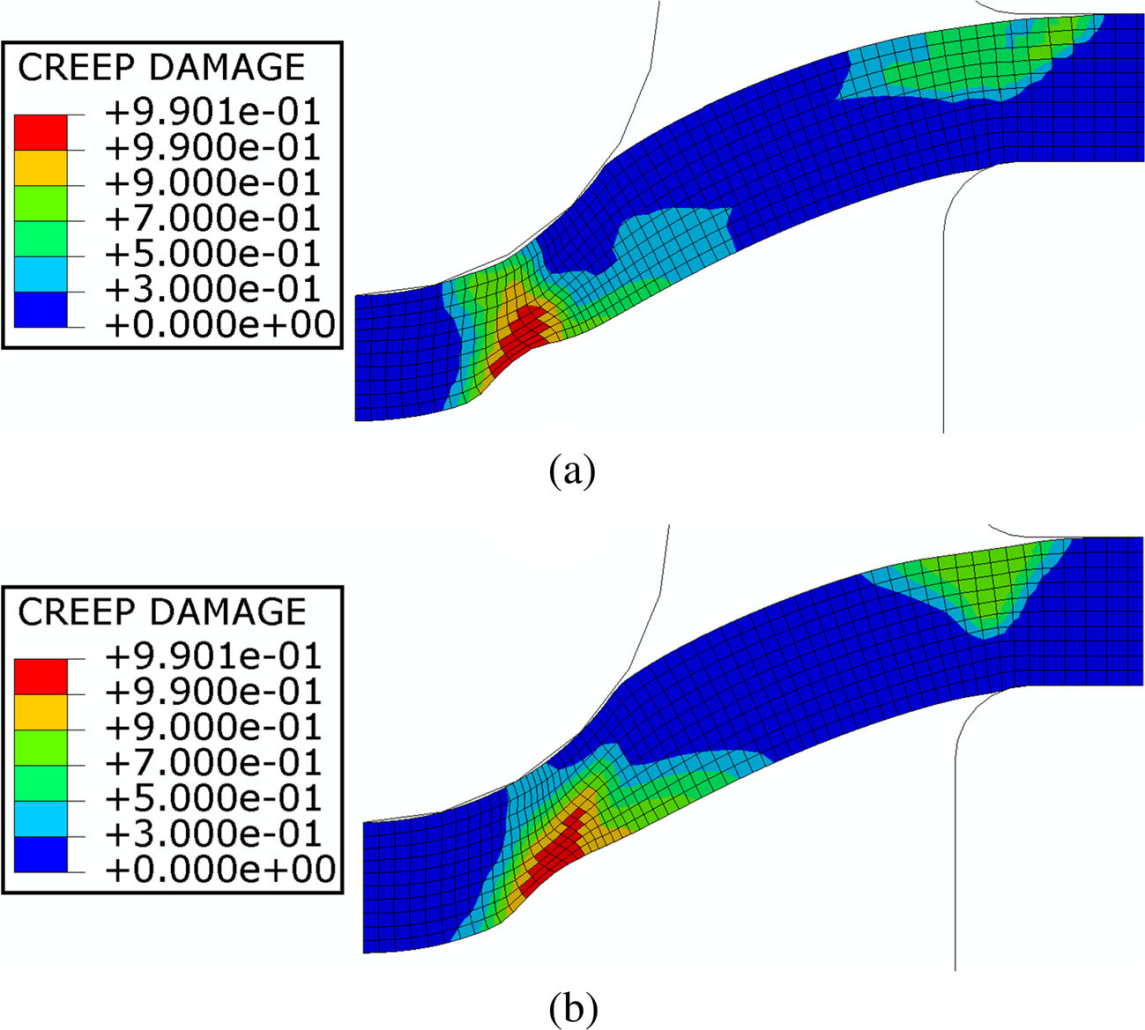
Contour plots of creep damage at 25*%* of the failure life for a load of 25*k* *g* (**a**) with and (**b**) without including the effects of initial plastic deformations on material creep behaviour

Figure 25 shows a comparison of contour plots of creep damage for a punch load of 25*k* *g*. These plots show the results obtained with the modified constitutive model at 25*%* of the failure life (462*h* *r* *s*) and those of the FE analysis without the inclusion of the plastic deformation effects at the same failure life fraction (42*h* *r* *s*). It should be noted that the high damage locations indicated in Fig. 25 are compatible with the region of the specimen where crack initiation and propagation was found during the interrupted tests discussed in “Small punch creep test results” (see Fig. 13). Indeed, crack initiation location suggested in Fig. 12(b) and the secondary crack location in Fig. 13(c) correlate well with the high damage region reported in both models.

The results of the FE calculations show that the peak damage location is, in both cases, near the bottom surface of the specimen and in line with the contact edge between the punch and the specimen (agreeing with previous observations from the literature [3, 43]). The region where peak damage is located, defined in the literature as the most critical location of the specimen [16, 28], exhibits a global creep enhancement. It is also noted that the region where the elements are fully damaged is larger when the plastic deformation effects are considered. The propagation of the high damage region is not identical in both cases as a result of different creep damage evolution properties (the inclusion of initial plasticity effects). When the effects of pre-straining are taken into account, the damage level in the region close to the clamps is lower than that obtained from the non-modified Liu-Murakami constitutive model, since the elements in that area exhibit a creep resistance effect and the damage rate evolution process becomes slower.

In spite of the larger high damage region found in the 25*%* of the failure time analyses, the simulation with the inclusion of the plastic deformation effects showed a global creep resistance. The global behaviour of the specimen is governed by the combination of creep resistance effects, occurring in most of the specimen for this load level, and creep enhancement effects, found in the critical region of the specimen. For the loads of 28 and 30*k* *g*, a creep resistance effect was also observed because *ϕ* and *ψ* are less than unity in the majority of the specimen, as shown in Figs. 22 and 23.

A comparison of the results obtained by using the modified and the original damage models with the experimental data is given in Table 6.

**Table 6:** Summary of failure times and MDRs calculated from experimental (Exp.), Liu-Murakami (no pre-straining effects, L-M) and modified Liu-Murakami (inclusion of pre-straining effects, Mod. L-M) results and associated errors

Load (*kg*)	Method	MDR (*m* *m*/*h* *r*)	*t*_*f*_(*h* *r* *s*)	Error MDR (*%*)	Error *t*_*f*_(*%*)
25	Exp.	4.46x 10^−4^	1068.38	-	-
—–	L-M	4.05x 10^−3^	170.94	208.07	84.00
—–	Mod. L-M	4.07x 10^−4^	1850.43	8.74	73.20
28	Exp.	1.29x 10^−3^	278.49	-	-
—–	L-M	1.56x 10^−2^	49.46	1109.30	82.24
—–	Mod. L-M	1.28x 10^−3^	497.85	0.78	78.77
30	Exp.	2.14x 10^−3^	176.09	-	-
—–	L-M	2.36x 10^−2^	29.35	1002.80	83.33
—–	Mod. L-M	2.66x 10^−3^	237.5	24.30	34.87

It is generally observed that estimations of both MDR and *t*_*f*_ are improved if initial plasticity effects are considered. While absolute errors are still high when the modified Liu-Murakami model is implemented (average of 62.28*%* compared to 83.19*%* for un-modified cases), it should be noted that failure lives are generally of the same order of magnitude as the experimental results. In SPCT analyses where the un-modified Liu-Murakami model is implemented, failure lives are very short (damage accumulation is much higher in the simulations where initial plasticity is not considered due to potential creep resistance effects being neglected). It is suspected that the overestimation of failure time is due to shift in dominance from creep deformation to creep crack growth in the SPCT. It can be seen from optical microscopy that cracks initiate early (2/3*t*_*f*_) for the 25*k* *g* load case. The modelling approach adopted at present does not allow for crack initiation and growth, hence the general over prediction of *t*_*f*_. The peak damage location was not affected by the initial plastic deformation but the direction of propagation of the high damage region was different from that obtained by the FE calculations without considering the effects of pre-straining. This is again due to the complex interaction between creep enhancement and creep resistant regions in the SPCT. Modelling conditions are in part verified by the excellent agreement noted between high damage regions in FEA model results and crack initiation locations found from interrupted SPCTs (see Figs. 11, 12 and 13).

## Concluding Remarks

Small specimen tests are gaining popularity in many industries (such as power generation and aerospace) due to their ability to estimate bulk material properties from very small amounts of sample material. Structural integrity assessments of in service components can be enhanced using these quasi-non destructive techniques as the overall component integrity is not impaired after sampling [51]. A limiting factor in the wider implementation of the SPCT however is the difficulty in interpreting test results and determining equivalent uniaxial behaviours.

In the present paper, the results of SPCTs (complete and interrupted) have been reported and the role of the initial, localised plastic pre-straining on the subsequent creep behaviour of a P91 steel at 600 ^∘^C has been investigated (creep enhancement or resistance).

- A modified Liu-Murakami creep damage model, in which the effect of the initial plasticity is accounted for, has been developed based on results for pre-strained uniaxial creep tests and the corresponding SPCT experimental results.
- Prior plastic deformation was found to significantly affect the creep curve of the material tested, and two parameters, *ϕ* and *ψ*, have been used to describe the variation of the minimum creep rate and failure time induced by pre-straining, respectively.
- Predictions of minimum displacement rates (MDRs) in SPCTs were vastly superior when FEA models included pre-straining effects.
- Some improvement was also noted for the prediction of time to failure, *t*_*f*_.

## Future Work

The present work has highlighted the critical importance of considering initial plasticity when analysing SPCT results. Accurate interpretation of the SPCT results will only be possible with this greater appreciation of the deformation behaviour of the sample.

- Future inverse methods must make some assumptions on the creep enhancement/resistance regions in the SPCT sample prior to performing inverse studies to determine creep material properties. Improvements to the prediction of the creep enhancement/resistance behaviour of a particular type of material (here high chrome steels) can be made by incorporating loading (stress) dependent terms in the *ϕ* and *ψ* functions.
- In order to avoid over prediction of *t*_*f*_, future models should take into account crack initiation and propgation.
- More data in the experimental region of large plastic strains of pre-strained uniaxial creep specimens would enhance the modified Liu and Murakami creep damage model in terms of degree of accuracy and reliability.
- The minimum creep strain rate and the time to rupture in uniaxial creep tests are linked by the Monkmann-Grant equation. The investigation of the effects of pre-strain on the parameters of such relation may lead to the identification of the link between *ϕ* and *ψ*.
- Since creep enhancement and creep resistance effects are microstructure-controlled, a microstructural investigation needs to be carried out.
- A useful improvement could consist of the characterisation of the effects of stick/slip contact regime between the punch and the specimen and the development of a technique to mitigate it, in order to increase test repeatability.
- Unified visco-elastic visco-plastic constitutive models could be superior alternatives to Liu and Murakami creep damage model for numerical simulation of small punch creep test.

## Appendix

In order to derive equation (2), the non-linear problem of load correction has to be split in three parts: elastic part of pre-straining, plastic part of pre-straining and elastic recovery upon unloading.

### Elastic Part of Pre-straining

*P*_*e**l*,*y**i**e**l**d*_ is the load during the elastic part of pre-straining that leads the specimen to yield, and it is given by equation (16), where *d*_*e**l*,*y**i**e**l**d*_ is the diameter of the effective section of the specimen at the elastic limit, defined by equation (17).

16 $$ P_{el,yield}=\sigma\pi\frac{{d_{el,yield}}^{2}}{4}  $$

17 $$ d_{el,yield}=d_{0}-\nu\epsilon_{el,yield}d_{0}=d_{0}(1-\nu\epsilon_{el,yield})  $$

In virtue of equation (17), *P*_*e**l*,*y**i**e**l**d*_ can be expressed as in equation (18).

18 $$ P_{el,yield}=\sigma\pi\frac{{d_{0}}^{2}}{4}{(1-\nu\epsilon_{el,yield})}^{2}  $$

### Plastic Part of Pre-straining

The load during the plastic part of pre-straining, *P*_*p**l*_, can be expressed as in equation (19), where *d*_*p**r**e*_ is the diameter of the effective section of the specimen due to pre-straining.

19 $$ P_{pl}=\sigma\pi\frac{{d_{pre}}^{2}}{4}  $$

State the constancy of specimen’s volume when plastic deformation takes place, equation (20) holds.

20 $$ {\Omega}_{pre}L_{pre}={\Omega}_{yield}L_{yield}  $$

Expressing equation (20) in the form of equation (21), it is possible to derive *d*_*p**r**e*_ as in equation (22).

21 $$ \pi\frac{{d_{pre}}^{2}}{4}L_{pre}=\pi\frac{{d_{el,yield}}^{2}}{4}L_{yield}  $$

22 $$ {d_{pre}}^{2}=d_{el,yield}^{2}\frac{L_{yield}}{L_{pre}}  $$

*L*_*p**r**e*_ and *L*_*y**i**e**l**d*_ are given in equation (23) and in equation (24), respectively, where *L*_0_ is the initial specimen gauge length.

23 $$ L_{pre}=L_{0}(1+\epsilon_{el,yield}+\epsilon_{p})  $$

24 $$ L_{yield}=L_{0}(1+\epsilon_{el,yield})  $$

Therefore, Ω_*p**r**e*_ can be obtained as in equation (25).

25 $$ {\Omega}_{pre}=\frac{L_{0}(1+\epsilon_{el,yield})}{L_{0}(1+\epsilon_{el,yield}+\epsilon_{p})}  $$

Ω_*e**l*,*y**i**e**l**d*_ is given in equation (26).

26 $$ {\Omega}_{el,yield}=\pi\frac{{d_{0}}^{2}}{4}(1-\nu\epsilon_{el,yield})^{2}  $$

Thus *P*_*p**l*_ can be expressed as in equation (27).

27 $$ P_{pl}=\sigma\pi\frac{{d_{0}}^{2}}{4}\frac{1+\epsilon_{el,yield}}{1+\epsilon_{el,yield}+\epsilon_{p}}  $$

### Elastic Recovery Upon Unloading

Upon unload partial recovery of *𝜖*_*e**l*,*y**i**e**l**d*_ to elastic strain, *𝜖*_*e**l*_, occurs, and the load *P* can be expressed in its final version as in equation (28).

28 $$ P=\sigma\pi\frac{{d_{0}}^{2}}{4}\frac{1+\epsilon_{el}}{1+\epsilon_{el}+\epsilon_{p}}  $$

Since *𝜖*_*e**l*_ is very small with respect to *𝜖*_*e**l*,*y**i**e**l**d*_, it can be neglected, and the load can be calculated as in equation (29).

29 $$ P\approx\sigma\pi\frac{{d_{0}}^{2}}{4}\frac{1}{1+\epsilon_{p}}  $$

## References

[1] Rouse JP, Cortellino F, Sun W, Hyde TH, Shingledecker J (2013). Small punch creep testing: review on modelling and data interpretation. Mater Sci Technol.

[2] Hyde TH, Sun W (2009). A novel, high sensitivity, small specimen creep test. Journal of Strain Analysis for Engineering Design.

[3] Evans M, Wang D (2008). The small punch creep test: some results from a numerical model. J Mater Sci.

[4] Evans RW, Evans M (2006). Numerical modelling of small disc creep test. Mater Sci Technol.

[5] CEN (2006). CWA 15627 Workshop agreement: Small punch test method for metallic materials.

[6] Hyde TH, Sun W, Ali BSM (2012) A small creep test specimen for use in determining uniaxial creep rupture data. IN: Matocha K, Hurst R, Sun W (eds) 2nd international conference SSTT determination of mechanical properties of materials by small punch and other miniature testing techniques. Ostrava (CZ), pp 261–270

[7] Blagoeva D, Li YZ, Hurst RC (2011). Qualification of p91 welds through small punch creep testing. J Nucl Mater.

[8] Gülçimen B, Hähner P (2013). Determination of creep properties of a p91 weldment by small punch testing and a new evaluation approach. Mater Sci Eng A.

[9] Holmstrom S, Auerkari P, Hurst R, Blagoeva D (2014). Using small punch test data to determine creep strain and strength reduction properties for heat affected zones. Mater Sci Technol.

[10] Baik JM, Kameda J, Buck O (1983). Small punch test evaluation of intergranular embrittlement of an alloy steel. Scr Metall.

[11] Dobeš F, Milička K (2008). Comparison of conventional and small punch creep tests of mechanically alloyed Al–C–O alloys. Mater Charact.

[12] Bagloeva D, Li YZ, Hurst RC (2011). Qualification of P91 welds through small punch creep testing. J Nucl Mater.

[13] Dymáček P (2016). Recent developments in small punch testing: applications at elevated temperatures. Theor Appl Fract Mech.

[14] Dymáček P, Dobes F (2012) Influence of atmosphere on small punch testing of p91 steel. In: Matocha K, Hurst R, Sun W (eds) 2nd international conference SSTT determination of mechanical properties of materials by small punch and other miniature testing techniques. Ostrava (CZ), pp 75–78

[15] Dymáček P, Milička K (2008). Small punch testing and its numerical simulations under constant deflection force conditions. Strength of Materials.

[16] Dymáček P, Milička K (2009). Creep small-punch testing and its numerical simulations. Mater Sci Eng A.

[17] Hurst RC (2005). Standardisation - a route to enhancing the acceptability of the small punch creep test. In: ECCC creep conference.

[18] Li Y, Sturm R (2008). Determination of creep properties from small punch test. ASME Conf Proc.

[19] Manahan MPS (1989). Mechanical behavior and swelling of tube scale from a pressurized water reactor steam generator using miniature specimens. J Nucl Mater.

[20] Milička K, Dobeš F (2006). Small punch testing of p91 steel. Int J Press Vessel Pip.

[21] Parker JD, Wilshire B (1992). Non-destructive life assessment of high temperature components and weldments. Int J Press Vessel Pip.

[22] Yang Z, Wang Z (2003). Relationship between strain and central deflection in small punch specimens. Int J Press Vessel Pip.

[23] Cortellino F (2015) Experimental and numerical investigation of small punch creep test. Ph.D Thesis, The Univeristy of Nottingham

[24] Cacciapuoti B, Sun W, McCartney DG (2016). A study on the evolution of the contact angle of small punch creep test of ductile materials. Int J Press Vessel Pip.

[25] Chakrabarty J (1970). A theory of stretch forming over hemispherical punch heads. Int J Mech Sci.

[26] Dobeš F, Milička K (2009). Application of creep small punch testing in assessment of creep lifetime. Materials Science and Engineering: A: Properties, Microstructure and Processing.

[27] Kobayashi KI, Kajihara I, Koyama H, Stratford GC (2010). Deformation and fracture mode during small punch creep tests. Journal of Solid Mechanics and Materials Engineering.

[28] Ma YW, Shim S, Yoon KB (2009). Assessment of power law creep constants of Gr91 steel using small punch creep tests. Fatigue and Fracture of Engineering Materials and Structures.

[29] Li DF, O’Dowd NP, Davies CM, Nikbin KM (2010). A review of the effect of prior inelastic deformation on high temperature mechanical response of engineering alloys. Int J Press Vessel Pip.

[30] Willis M, McDonaugh-Smith A, Hales R (1999). Prestrain effects on creep ductility of a 316 stainless steel light forging. Int J Press Vessel Pip.

[31] Wilshire B, Willis M (2004). Mechanisms of strain accumulation and damage development during creep of prestrained 316 stainless steels. Metall Mater Trans A.

[32] Wilshire B, Palmer CJ (2004). Strain accumulation during dislocation creep of prestrained copper. Mater Sci Eng A.

[33] Hyde TH (1986). Anomalous creep behaviour of 316 stainless steel at 550∘C. High Temp Technol.

[34] Hyde TH (1997). Creep of 316 stainless steel at 550 and 600∘C and the effects of short duration overloads on creep at 550∘C. Materials at High Temperatures.

[35] Liu Y, Murakami S (1998). Damage localization of conventional creep damage models and proposition of a new model for creep damage analysis. JSME International Journal Series A.

[36] Saad AA, Hyde TH, Sun W, Hyde CJ, Tanner DWJ (2013). Characterization of viscoplasticity behaviour of p91 and p92 power plant steels. Int J Press Vessel Pip.

[37] Cortellino F, Chen R, Sun W, Hyde TH (2014) A microscopic investigation on the failure mechanisms of small punch creep test of a p91 steel at 873 k. In: Matocha K, Hurst R, Sun W (eds) 2nd international conference SSTT determination of mechanical properties of materials by small punch and other miniature testing techniques. Graz (Austria), pp 260–269

[38] Tai K, Endo T (1993). Effect of pre-creep on the succeeding creep behavior of a 2.25Cr-1Mo steel. Scripta Metallurgica et Materialia.

[39] Kobayashi M, Koyama H, Stratford GC, Tabuchi M (2012). Kaneko the influence of both testing environment and fillet radius of the die holder on the rupture life of small punch creep tests. Journal of Solid Mechanics and Materials Engineering.

[40] Cortellino F, Sun W, Hyde TH (2016). On the effects of friction modelling on small punch creep test responses: a numerical investigation. The Journal of Strain Analysis for Engineering Design.

[41] Dymáček P, Seitl S, Milička K, Dobeš F (2010). Influence of friction on stress and strain distributions in small punch creep test models. Key Eng Mater.

[42] Hyde TH, Stoyanov M, Sun W, Hyde CJ (2010). On the interpretation of results from small punch creep tests. The Journal of Strain Analysis for Engineering Design.

[43] Ling X, Zheng Y, You Y, Chen Y (2007). Creep damage in small punch creep specimens of Type 304 stainless steel. Int J Press Vessel Pip.

[44] Zhou Z, Zheng Y, Ling X, Hu R, Zhou J (2010). A study on influence factors of small punch creep test by experimental investigation and finite element analysis. Mater Sci Eng A.

[45] Cortellino F, Sun W, Hyde TH, Shingledecker J (2014). The effects of geometrical inaccuracies of the experimental set-up on small punch creep test results. The Journal of Strain Analysis for Engineering Design.

[46] Hutchinson JW (1983). Constitutive behavior and crack tip fields for materials undergoing creep-constrained grain boundary cavitation. Acta Metallurgica.

[47] Riedel H (1987) Fracture at high temperatures. Springer

[48] Lamaitre J, Chaboche JL (2000) Mechanics of solid materials. Cambridge University Press

[49] ABAQUS (2010) Theory manual. Dassault SystÈMes Simulia Corp

[50] Lin FH, Tseng AA (1998). A finite element analysis of elasto-plastic contact problems in metal forming. Mater Des.

[51] Rouse JP, Sun W, Hyde TH (2013). The effects of scoop sampling on the creep behaviour of power plant straight pipes. Journal of Srain Analysis for Engineering Design.

